# Risk of adverse outcomes associated with mirtazapine compared to sertraline use among older people living in long-term care facilities

**DOI:** 10.1093/ageing/afaf074

**Published:** 2025-04-06

**Authors:** Georgina A Hughes, Maria C Inacio, Debra Rowett, Gillian E Caughey, Tracy Air, Catherine E Lang, Megan Corlis, Janet K Sluggett

**Affiliations:** University of South Australia, UniSA Clinical & Health Sciences, Adelaide, South Australia, Australia; Registry of Senior Australians Research Centre, South Australian Health and Medical Research Institute, Adelaide, South Australia, Australia; Registry of Senior Australians Research Centre, Caring Futures Institute, College of Nursing and Health Sciences, Flinders University, Bedford Park, South Australia, Australia; University of South Australia, UniSA Allied Health & Human Performance, Adelaide, South Australia, Australia; Drug and Therapeutics Information Service, Southern Adelaide Local Health Network, Adelaide, South Australia, Australia; Registry of Senior Australians Research Centre, South Australian Health and Medical Research Institute, Adelaide, South Australia, Australia; Registry of Senior Australians Research Centre, Caring Futures Institute, College of Nursing and Health Sciences, Flinders University, Bedford Park, South Australia, Australia; University of South Australia, UniSA Allied Health & Human Performance, Adelaide, South Australia, Australia; Registry of Senior Australians Research Centre, South Australian Health and Medical Research Institute, Adelaide, South Australia, Australia; Registry of Senior Australians Research Centre, South Australian Health and Medical Research Institute, Adelaide, South Australia, Australia; Australian Nursing & Midwifery Federation SA Branch, Adelaide, South Australia, Australia; Registry of Senior Australians Research Centre, South Australian Health and Medical Research Institute, Adelaide, South Australia, Australia; University of South Australia, UniSA Allied Health & Human Performance, Adelaide, South Australia, Australia

**Keywords:** antidepressant, mirtazapine, sertraline, safety, long-term care, older people

## Abstract

**Background:**

Antidepressants are used by 60% of residents of long-term care facilities (LTCFs). Mirtazapine and sertraline are the most commonly used antidepressants, despite little safety information for their use in LTCFs.

**Objective:**

To investigate risk of adverse outcomes (falls, fractures, cardiovascular-, dementia-, and delirium-related hospitalisations, all-cause mortality) associated with mirtazapine compared to sertraline use post-LTCF entry.

**Design:**

Active new user retrospective cohort study.

**Subjects:**

Individuals aged 65–105 years entering LTCFs in three Australian states during 1 January 2015 to 31 October 2018, who initiated mirtazapine or sertraline ≤60 days post-LTCF entry, with follow-up to 31 December 2019.

**Methods:**

The inverse probability of treatment weighting of individuals’ propensity scores was used to adjust Cox and Fine–Gray regression models to estimate the risk of outcomes of interest associated with mirtazapine compared to sertraline use in LTCFs. Weighted (adjusted) hazard ratios (aHRs), subdistribution hazard ratios and 95% confidence intervals (95% CIs) are presented.

**Results:**

A total of 5409 residents initiated mirtazapine (71%, *n* = 3837) or sertraline (29%, *n* = 1572) post-LTCF entry. After weighting, mirtazapine was associated with a higher risk of mortality (aHR 1.16, 95% CI 1.05–1.29) compared to sertraline. The risk of falls and fractures within 90 days was not statistically significantly different between the groups but was lower in mirtazapine users thereafter. No differences in risk of cardiovascular-, dementia- or delirium-related hospitalisations were observed.

**Conclusions:**

Compared to sertraline, mirtazapine use is associated with a higher risk of mortality and, after 90 days of use, a lower risk of falls and fractures. This risk of harm should be balanced with limited evidence for effectiveness when considering antidepressant therapy in LTCFs.

## Key Points

- Risk of adverse harms in residents of long-term care facilities (LTCFs) using mirtazapine or sertraline were examined.
- Over 5 years, incident mirtazapine use was associated with a 16% higher risk of all-cause mortality compared to sertraline.
- Risks of falls and fractures did not differ in the first 90 days of use but were lower in mirtazapine users thereafter.
- There were no differences in the risk of cardiovascular-, dementia- or delirium-related hospitalisations.
- Risk of harm associated with mirtazapine use compared to sertraline warrants individualised treatment approaches in LTCFs.

## Introduction

A total of 6 in 10 residents of long-term care facilities (LTCFs) use an antidepressant [1, 2]. Mirtazapine, a sedating tetracyclic antidepressant, and sertraline, a selective serotonin reuptake inhibitor (SSRI), are the most common antidepressants used in LTCFs [1]. Mirtazapine use more than doubled between 2006 and 2019 [1] and is commonly initiated on LTCF entry [3]. Mirtazapine is indicated for moderate–severe major depression [4]; however, potential off-label use for mild depressive symptoms, behaviour, mood or sleep changes (which are common following LTCF entry) has been reported [3, 5–7]. Substitution of antipsychotics with sedating antidepressants, including mirtazapine, has been described in response to antipsychotic regulations in LTCFs [2]. High rates of mirtazapine use are concerning given limited evidence to support its safe and effective use among residents of LTCFs [8] who often experience cognitive decline [9].

Systematic reviews of antidepressant safety and effectiveness among older people have used various study designs and report conflicting findings with evidence for efficacy being modest [10–13]. Three randomised controlled trials (RCTs) among older people [14–16] had small study sizes (<300 participants) and short follow-ups (16 weeks maximum), used comparators not commonly used for treating depression in LTCFs (i.e. amitriptyline, trazodone, paroxetine [1, 5]) and were subject to sponsorship bias with mirtazapine [14–16]. Two RCTs found mirtazapine is not effective for depression or agitation in dementia [17, 18].

Observational studies report an association between mirtazapine use and an increased risk of falls, fractures, cardiovascular events or mortality among older people [19–21]. Three studies have investigated mirtazapine safety and effectiveness specifically in LTCFs, including an open-label study (*n* = 115 residents aged ≥70 years with depression and without severe cognitive impairment) [22] and two secondary analyses (*n* = 49–119) [23, 24]. All three studies were subject to sponsorship bias, limited follow-up (12 weeks) and did not employ comparator arms, and approximately one-third of the participants discontinued mirtazapine [22–24]. Of these open-label studies, only one reported a statistically significant reduction in an assessed depression score and the clinical significance is unclear [22]. Urinary tract infections, injury and falls were reported in up to one-quarter of the individuals [22, 23].

Given increasing mirtazapine use, together with a paucity of effectiveness and safety information for residents of LTCFs, leveraging real-world data to examine the risk for adverse outcomes associated with mirtazapine is needed to inform safe antidepressant use in LTCFs. This study investigated the risk of adverse outcomes (i.e. falls, fractures, cardiovascular events, dementia- and delirium-related hospitalisations, and all-cause mortality) among older people initiating mirtazapine following LTCF entry, compared to sertraline. Sertraline was selected as an active comparator as both agents are first-line pharmacological therapies for moderate–severe major depression in Australia [4, 25].

## Methods

### Study design and data source

An active new user propensity score-weighted retrospective cohort study was conducted using de-identified data from the Registry of Senior Australians (ROSA) National Historical Cohort. ROSA, a national data platform, contains integrated health and aged care service utilisation, medical, pharmaceutical, sociodemographic and mortality information for older people (≥65 years) who were assessed for eligibility or accessed government-subsidised aged care services from 2002 onwards [26]. ROSA’s core datasets include those within the Australian Institute of Health and Welfare (AIHW) National Aged Care Data Clearinghouse (including the National Death Index), Medicare Benefits Schedule (MBS) and Pharmaceutical Benefits Scheme (PBS) claims records. The PBS dataset includes details of claims for the supply of subsidised medicines in Australia, coded using PBS item codes and mapped to World Health Organization Anatomical Therapeutic Chemical (ATC) codes [27]. Hospital and emergency department presentation datasets in public and private hospitals in New South Wales (NSW), Queensland (QLD) and Victoria (VIC) are also linked to ROSA.

### Study setting and cohort

**Figure 1:**
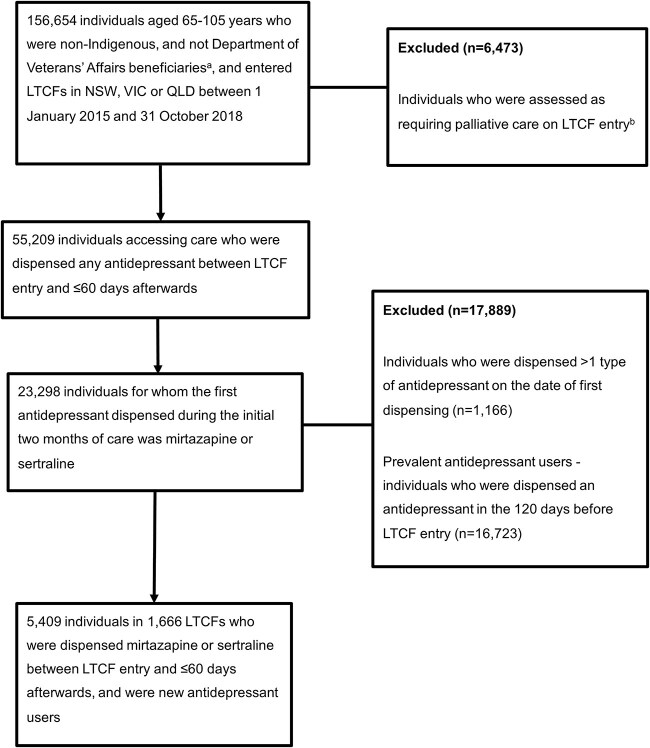
Flow chart for study cohort selection. ^a^There were 632 individuals missing information for Department of Veterans’ Affairs beneficiary. ^b^There were 10 724 individuals missing information for palliative care requirements on LTCF entry.

Non-Indigenous individuals aged 65–105 years who entered a LTCF in NSW, QLD or VIC between 1 January 2015 and 31 October 2018 and initiated mirtazapine or sertraline between LTCF entry and ≤60 days after (while in care) were included. Among these individuals, new users were defined as those who were not dispensed an antidepressant in the 120 days before LTCF entry (Supplementary Figure 1). Individuals were excluded if they were codispensed another antidepressant on the same day, were Department of Veterans’ Affairs beneficiaries (who can have different MBS-subsidised service access and influence outcome ascertainment) or if they required palliative care on entry. Specific leadership, governance and ethical approvals are required for analysis of Indigenous individuals’ records and were not part of this study. The index date was the date of first mirtazapine or sertraline dispensing. If individuals entered a LTCF permanently within 1 day of respite care, consecutive (±1 day) records of respite care were linked to determine the LTCF entry date. Residents did not enter a LTCF permanently after 31 December 2019. The cohort included 5409 individuals (Figure 1).

### Exposure of interest

Mirtazapine (ATC N06AX11) or sertraline (ATC N06AB06) dispensings were identified from PBS claims.

### Outcomes of interest

The outcomes of interest were falls, fractures, cardiovascular events, dementia- and delirium-related hospitalisations and all-cause mortality (Supplementary Table 1). Falls, fractures and dementia- and delirium-related hospitalisations were determined using ROSA’s Outcome Monitoring System definitions [28]. Cardiovascular events included stroke, myocardial infarction or transient ischemic attack as the principal diagnosis for a hospitalisation or emergency department presentation. A sensitivity analysis also included these death diagnoses in accordance with previous studies using claims data [29, 30].

In the primary analysis, all individuals had the opportunity for a minimum of 1-year follow-up and were censored at discontinuation of the index antidepressant or dispensing of another antidepressant {median time to antidepressant discontinuation was 164 days [interquartile range (IQR) 50–442] for mirtazapine and 151 days [IQR 50–443] for sertraline users}, LTCF exit, death or 31 December 2019, whichever occurred first. To determine continuity of exposure, antidepressant discontinuation was defined by a gap in dispensing of two prescription durations after the date the current supply ended [31]. The prescription duration was calculated as the period that 75% of the cohort received a repeat supply in 2015 [32].

### Covariates

Potential confounders included in propensity score calculations included individual and medicine-, service- and facility-related factors. Individual factors were determined using aged care eligibility or entry to LTCF assessments and included age at LTCF entry, sex, marital status, preferred language, country of birth, comorbidity score at LTCF entry, levels of assisted daily living, behavioural daily living and complex healthcare needs and health conditions [depression, dementia, diabetes, osteoporosis, malnutrition and history of falls, fractures, cardiovascular events (stroke, myocardial infarction, transient ischaemic attack) or delirium]. RxRisk-V, the medication-based comorbidity score, determined the comorbidity score and identified osteoporosis, dementia, diabetes and malnutrition [33]. Medicine-related factors included the number of unique medicines (by ATC code) dispensed in the year before LTCF entry and the use of specific medicine classes ≤120 days before LTCF entry, including antipsychotic, benzodiazepine or zopiclone, opioid, bisphosphonate, lithium, antihypertensive, lipid-lowering, anticoagulant, antiplatelet, anti-arrhythmic, antianginal, loop diuretic, aldosterone antagonist, other heart failure medicine or medicine for Parkinson’s disease. System- and facility-related factors included the number of general medical practitioner visits (MBS groups A01, A02, A35), emergency department presentations and unplanned hospitalisations in the year before LTCF entry, LTCF provider type, remoteness and state of residence. Other covariates included time to antidepressant initiation post LTCF-entry (days) and year of study entry.

### Statistical analysis

The cohort was characterised using descriptive statistics. Logistic regression determined the individuals’ probability of receiving the studied treatment (a propensity score) using complete case analysis (*n* = 182, 3.4% missing data). Inverse probability of treatment weighting (IPTW) was used to estimate the average treatment effect at the population level [34]. The balance of weighted propensity scores was assessed using standardised differences after weighting [34].

Time to each outcome for mirtazapine and sertraline users in the weighted population was determined by Kaplan–Meier (for all-cause mortality) and cumulative incidence function plots accounting for the competing risk of death (for all other outcomes). Cox proportional hazards models estimated crude and weighted (adjusted) hazard ratios (aHRs) and 95% Wald confidence intervals (CIs) for all-cause mortality and as an additional analysis for all other outcomes. Fine–Gray competing risk regression models estimated adjusted subdistribution hazard ratios (aSHRs) and 95% CIs for all outcomes other than mortality [35]. To account for nonindependence in observations caused by IPTW-weighting, robust variance estimation (by resident and LTCF) was used in all models. Proportional hazards assumptions were assessed and where violated (for falls and fractures) time-dependent estimates were estimated. Visual examination of these cumulative incidence function plots identified time periods (i.e. ≤90 and >90 days). The Bonferroni correction was used to correct for multiple hypothesis testing in the main analysis (a priori *P* =0.006).

A sensitivity analysis using an intention-to-treat approach, which followed residents until LTCF exit, death or 31 December 2019, whichever occurred first, was conducted. The risk of each outcome within 90, 365 and 730 days was determined. Principal causes of death (recorded by International Classification of Diseases Tenth Revision, Australian Modification codes) during follow-up were described. SAS, version 9.4 (SAS Institute Inc., Cary, NC, USA) and Stata v18.0 (StataCorm, College Station, TX, USA) were used for analysis.

#### Ethics approval

This study was approved by the University of South Australia (ref: 200489), AIHW (ref: EO2022/4/1376), South Australian Department for Health & Wellbeing (ref: HREC/18/SAH/90) and NSW Population & Health Services (ref: 2019/ETH12028) Research Ethics Committees.

**Table 1:** Baseline characteristics of cohort before and after IPTW and standardised mean differences (SD) after weighting.

Baseline characteristic (*n*,% unless otherwise stated)	Overall	Crude		Weighted^a^		Weighted SD
		Mirtazapine	Sertraline	Mirtazapine	Sertraline	
Number of residents	5409	3837 (70.9)	1572 (29.1)	3711	1517	
Year of study entry						
2015	1300 (24.0)	899 (23.4)	401 (25.5)	923 (24.9)	364 (24.0)	0.002
2016	1397 (25.8)	973 (25.4)	424 (27.0)	964 (26.0)	410 (27.0)	
2017	1444 (26.7)	1026 (26.7)	418 (26.6)	973 (26.2)	409 (27.0)	
2018	1268 (23.4)	939 (24.5)	329 (20.9)	851 (22.9)	333 (22.0)	
Sex (female)	3047 (56.3)	2188 (57.0)	859 (54.6)	2101 (56.6)	860 (56.7)	−0.002
Age at LTCF entry (median, IQR)	84 (79–89)	84 (79–89)	84 (78–88)	84 (79–89)	84 (79–89)	−0.005
Born in Australia^b^	3460 (64.0)	2405 (62.7)	1055 (67.1)	2382 (64.2)	969 (63.9)	0.006
Primary language is English^b^	4752 (87.9)	3338 (87.0)	1414 (89.9)	3287 (88.6)	1341 (88.4)	0.004
RxRisk-V comorbidity score on LTCF entry (median, IQR)^c^	4 (2–6)	4 (2–6)	4 (2–6)	4 (2–6)	4 (2–6)	−0.010
Health conditions^b^						
Dementia diagnosis	2812 (52.0)	2012 (52.4)	800 (50.9)	1940 (52.3)	794 (52.3)	0.002
Depression diagnosis	3295 (60.9)	2275 (59.3)	1020 (64.9)	2271 (61.2)	924 (60.9)	−0.006
History of falls	1255 (23.2)	883 (23.0)	372 (23.7)	858 (23.1)	350 (23.1)	−0.002
History of fractures	719 (13.3)	519 (13.5)	200 (12.7)	501 (13.5)	209 (13.8)	0.009
History of transient ischemic attack	170 (3.1)	114 (3.0)	56 (3.6)	119 (3.2)	49 (3.2)	0.000
History of stroke	1106 (20.4)	755 (19.7)	351 (22.3)	760 (20.5)	310 (20.4)	−0.001
History of myocardial infarction	226 (4.2)	151 (3.9)	75 (4.8)	159 (4.3)	68 (4.5)	0.009
History of delirium	404 (7.5)	298 (7.8)	106 (6.7)	278 (7.5)	118 (7.8)	0.011
Diabetes	1233 (22.8)	861 (22.4)	372 (23.7)	846 (22.8)	347 (22.9)	0.002
Osteoporosis	1214 (22.4)	872 (22.7)	342 (21.8)	840 (22.6)	346 (22.8)	0.004
Malnutrition	119 (2.2)	91 (2.4)	28 (1.8)	81 (2.2)	35 (2.3)	0.008
Activities of daily living level^b^						
None	24 (0.4)	18 (0.5)	6 (0.4)	17 (0.5)	8 (0.5)	[ref]
Low	678 (12.5)	482 (12.6)	196 (12.5)	469 (12.6)	193 (12.7)	0.002
Medium	1692 (31.3)	1183 (30.8)	509 (32.4)	1169 (31.5)	484 (31.9)	0.008
High	2975 (55.0)	2125 (55.4)	850 (54.1)	2057 (55.4)	833 (54.9)	−0.010
Behavioural daily living level^b^						
None	173 (3.2)	133 (3.5)	40 (2.5)	122 (3.3)	51 (3.4)	[ref]
Low	492 (9.1)	340 (8.9)	152 (9.7)	341 (9.2)	137 (9.0)	−0.006
Medium	1188 (22.0)	828 (21.6)	360 (22.9)	821 (22.1)	340 (22.4)	0.008
High	3516 (65.0)	2507 (65.3)	1009 (64.2)	2427 (65.4)	989 (65.2)	−0.004
Complex health care level^b^						
None	97 (1.8)	64 (1.7)	33 (2.1)	68 (1.8)	29 (1.9)	[ref]
Low	1201 (22.2)	835 (21.8)	366 (23.3)	823 (22.2)	335 (22.1)	−0.002
Medium	1636 (30.2)	1156 (30.1)	480 (30.5)	1134 (30.6)	463 (30.5)	0.000
High	2435 (45.0)	1753 (45.7)	682 (43.4)	1687 (45.5)	689 (45.4)	−0.001
Marital status^b^						
Widowed	2181 (40.3)	1556 (40.6)	625 (39.8)	1530 (41.2)	628 (41.4)	0.004
Married or de facto	2157 (39.9)	1541 (40.2)	616 (39.2)	1505 (40.6)	616 (40.6)	0.002
Divorced	521 (9.6)	363 (9.5)	158 (10.1)	366 (9.9)	147 (9.7)	−0.005
Separated	48 (0.9)	35 (0.9)	13 (0.8)	34 (0.9)	12 (0.8)	−0.010
Never married	399 (7.4)	273 (7.1)	126 (8.0)	278 (7.5)	113 (7.4)	[ref]
State of residence						
NSW	2320 (42.9)	1648 (43.0)	672 (42.8)	1589 (42.8)	649 (42.8)	−0.000
VIC	1855 (34.3)	1312 (34.2)	543 (34.5)	1274 (34.3)	526 (34.7)	[ref]
QLD	1234 (22.8)	877 (22.9)	357 (22.7)	848 (22.9)	342 (22.5)	−0.007
Remoteness of residence^b^						
Major cities	3829 (70.8)	2834 (73.9)	995 (63.3)	2637 (71.1)	1079 (71.1)	−0.001
Outside major cities	1567 (29.0)	996 (26.0)	571 (36.3)	1074 (28.9)	438 (28.9)	
Type of LTCF						
Private	2415 (44.6)	1744 (45.5)	671 (42.7)	1661 (44.8)	681 (44.9)	[ref]
Not for profit	2717 (50.2)	1918 (50.0)	799 (50.8)	1865 (50.3)	759 (50.0)	−0.004
Government	277 (5.1)	175 (4.6)	102 (6.5)	186 (5.0)	76 (5.0)	0.000
Number of GP visits in the year before LTCF entry (median, IQR)	10 (5–17)	11 (6–17)	10 (5–17)	10 (6–17)	10 (5–18)	−0.007
Number of ED presentations in the year before LTCF entry (median, IQR)	1 (1–3)	1 (1–3)	1 (1–3)	1 (1–3)	1 (1–3)	0.006
Number of unplanned hospitalisations in the year before LTCF entry (median, IQR)	1 (1–2)	1 (1–2)	1 (1–2)	1 (1–2)	1 (1–2)	0.002
Number of unique medicines dispensed in the year before LTCF entry (median, IQR)^c^	9 (5–14)	9 (6–14)	9 (5–14)	9 (6–14)	9 (5–14)	−0.018
Specific classes of medicines dispensed in the 120 days before LTCF entry						
Antipsychotic	567 (10.5)	425 (11.1)	142 (9.0)	386 (10.4)	154 (10.2)	−0.008
Benzodiazepine or zopiclone	989 (18.3)	768 (20.0)	221 (14.1)	683 (18.4)	278 (18.3)	−0.001
Medicine for Parkinson’s disease	260 (4.8)	192 (5.0)	68 (4.3)	179 (4.8)	78 (5.1)	0.014
Opioid	1271 (23.5)	940 (24.5)	331 (21.1)	870 (23.4)	361 (23.8)	0.008
Bisphosphonate	311 (5.7)	234 (6.1)	77 (4.9)	214 (5.8)	91 (6.0)	0.009
Antiplatelet	920 (17.0)	640 (16.7)	280 (17.8)	634 (17.1)	264 (17.4)	0.009
Anticoagulant	1129 (20.9)	819 (21.3)	310 (19.7)	777 (20.9)	320 (21.1)	0.004
Antianginal	427 (7.9)	300 (7.8)	127 (8.1)	297 (8.0)	124 (8.2)	0.006
Anti-arrhythmic	504 (9.3)	369 (9.6)	135 (8.6)	351 (9.5)	140 (9.2)	−0.007
Lipid lowering	2040 (37.7)	1445 (37.7)	595 (37.8)	1401 (37.8)	577 (38.0)	0.006
Antihypertensive	3229 (59.7)	2316 (60.4)	913 (58.1)	2222 (59.9)	907 (59.8)	−0.001
Furosemide	1061 (19.6)	769 (20.0)	292 (18.6)	731 (19.7)	300 (19.8)	0.001
Aldosterone antagonist	289 (5.3)	211 (5.5)	78 (5.0)	195 (5.3)	80 (5.3)	0.001
Other medicines for heart failure	9 (0.2)	N/A	N/A	N/A	N/A	−0.009
Time to antidepressant initiation (days) [median, IQR]	18 (3–37)	17 (3–37)	20 (4–39)	18 (3–38)	19 (3–37)	−0.004

SD, standardised mean differences; N/A, not available (due to low counts); GP, general practitioner; ED, emergency department.

Lithium dispensed in the 4 months before LTCF entry was assessed but not reported due to low counts. There were 432 (8.0%) of individuals without PBS medicines dispensed 6 months before LTCF entry and 595 (11.0%) 4 months before LTCF entry.

^a^IPTW cohort does not include *n* = 182 individuals with missing data for determining propensity scores (complete case analysis).

^b^Missing data *n* (%): country of birth 20 (0.4), primary language 28 (0.5), entry to care assessment 40 (0.7), aged care eligibility assessment 268 (5.0), remoteness 13 (0.2) and marital status 103 (1.9).

^c^Number of unique medicines and comorbidity score do not include antidepressants.

## Results

There were 5409 individuals from 1666 LTCFs who initiated mirtazapine (*n* = 3837, 70.9%) or sertraline (*n* = 1572, 29.1%) within ≤60 days of LTCF entry, with a median age of 84 years (IQR 79–89). Table 1 shows the IPTW cohorts and standardised differences. Residents were followed up to 5 years, with overall median follow-up of 258 days (IQR 70–634), 252 days (IQR 68–622) among mirtazapine (*n* = 3711) and 279 days (IQR 72–672) among sertraline (*n* = 1517) users in the IPTW cohort (*n* = 5228).

### Falls and fractures

**Figure 2:**
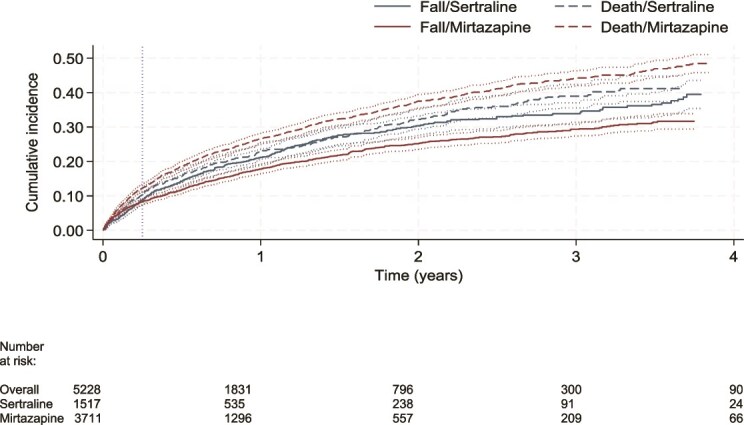
Cumulative incidence of falls and competing risk of death among the weighted cohorts, by antidepressant type. Cumulative incidence of falls, and death as the competing event, is plotted. Dotted vertical line at 90 days for time-varying effects. Plot truncated to 4 years due to low numbers at risk thereafter. Numbers at risk of the IPTW cohort are listed by exposure group at bottom of plot.

The cumulative incidence of falls, accounting for the competing risk of death, was 34.5% (95% CI 31.5–37.5) for mirtazapine and 43.0% (95% CI 35.0–50.8) for sertraline users (Figure 2). The cumulative incidence of fractures was 18.5% (95% CI 15.3–21.9) in mirtazapine and 25.7% (95% CI 18.6–33.4) in sertraline users (Supplementary Figure 2). There were no statistically significant differences in the risk of falls (aSHR 0.99, 95% CI 0.80–1.23, *P* =0.938) or fractures (aSHR 0.93, 95% CI 0.66–1.31, *P* = 0.673) between mirtazapine and sertraline users in the first 90 days. There was a lower risk of falls (aSHR 0.74, 95% CI 0.63–0.87, *P* <0.001) and fractures (aSHR 0.64, 95% CI 0.52–0.80, *P* <0.001) in mirtazapine users after 90 days, compared to sertraline (Table 2).

**Table 2:** Number of individuals experiencing adverse outcomes and associated risk among residents initiating mirtazapine versus sertraline.

Outcome	Crude (*n*,%)		Weighted^a^ (*n*,%)		Weighted aHR (95% CI, *P*)^a^	aSHR (95% CI, *P*)*
	Mirtazapine (*n* = 3837)	Sertraline (*n* = 1572)	Mirtazapine (*n* = 3711)	Sertraline (*n* = 1517)		
Fall						
Overall	775 (20.2)	373 (23.7)	746 (20.1)	363 (23.9)	0.86 (0.75–0.97), 0.018	**0.82 (0.72–0.94), 0.003**
≤90 days	297 (7.7)	126 (8.0)	285 (7.7)	118 (7.8)	1.01 (0.81–1.25), 0.961	0.99 (0.80–1.23), 0.938
>90 days	478 (12.5)	247 (15.7)	461 (12.4)	245 (16.2)	**0.78 (0.67–0.92), 0.003**	**0.74 (0.63–0.87), <0.001**
Fracture						
Overall	361 (9.4)	193 (12.3)	343 (9.2)	194 (12.8)	**0.74 (0.62–0.89), 0.001**	**0.71 (0.59–0.85), <0.001**
≤90 days	109 (2.8)	49 (3.1)	104 (2.8)	46 (3.0)	0.94 (0.67–1.34), 0.739	0.93 (0.66–1.31), 0.673
>90 days	252 (6.6)	144 (9.2)	239 (6.4)	148 (9.8)	**0.68 (0.55–0.84), <0.001**	**0.64 (0.52–0.80), <0.001**
Cardiovascular event						
Main analysis	187 (4.9)	79 (5.0)	183 (4.9)	71 (4.7)	1.11 (0.84–1.45), 0.459	1.06 (0.81–1.39), 0.684
Sensitivity analysis^**b**^	316 (8.2)	127 (8.1)	314 (8.5)	120 (7.9)	1.12 (0.91–1.39), 0.290	1.07 (0.87–1.33), 0.513
Dementia	126 (3.3)	50 (3.2)	124 (3.3)	51 (3.4)	1.03 (0.74–1.44), 0.862	1.00 (0.71–1.39), 0.975
Delirium	212 (5.5)	72 (4.6)	195 (5.4)	68 (4.5)	1.22 (0.92–1.62), 0.172	1.17 (0.88–1.56), 0.267
All-cause mortality	1471 (38.3)	556 (35.4)	1429 (38.5)	531 (35.0)	**1.16 (1.05–1.29), 0.004**	N/A

N/A, not applicable. Statistically significant results are shown in bold.

^a^IPTW cohort does not include *n* = 182 individuals with missing data for determining propensity scores (complete case analysis).

^b^Sensitivity analysis for ascertaining cardiovascular events also included cardiovascular event death diagnoses.

### Cardiovascular events

The cumulative incidence of cardiovascular events, accounting for the competing risk of death was 9.0% (95% CI 7.2–11.1) for mirtazapine and 8.6% (95% CI 6.1–11.5) for sertraline users (Supplementary Figure 3). There was no statistically significant difference in risk of cardiovascular events between mirtazapine and sertraline (aSHR 1.06, 95% CI 0.81–1.39, *P* =0.684).

### Dementia- and delirium-related hospitalisations

The cumulative incidence of dementia-related hospitalisations was 4.5% (95% CI 3.7–5.5) for mirtazapine, and 5.3% (95% CI 3.9–7.0) for sertraline users, accounting for the competing risk of death (Supplementary Figure 4). The cumulative incidence of delirium-related hospitalisations was 8.8% (95% CI 7.2–10.5) for mirtazapine and 7.2% (95% CI 5.4–9.2) among sertraline users (Supplementary Figure 5). There was no statistically significant difference in risk of dementia-related (aSHR 1.00, 95% CI 0.71–1.39, *P* =0.975) or delirium-related (aSHR 1.17, 95% CI 0.88–1.56, *P* =0.267) hospitalisations between mirtazapine and sertraline users.

### All-cause mortality

**Figure 3:**
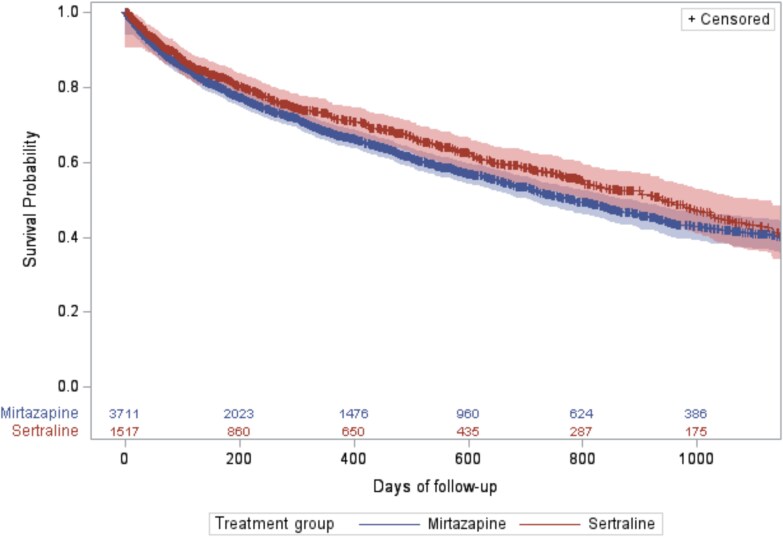
Kaplan–Meier survival plot with 95% confidence bands for time to all-cause mortality among the weighted cohorts, by antidepressant type. Plot truncated to 3 years due to low numbers at risk thereafter. Numbers at risk of the IPTW cohort are listed by exposure group at bottom of plot.

The proportion of people who died during follow-up was 38.5% (*n* = 1429) among mirtazapine and 35.0% (*n* = 531) among sertraline users. There was a higher risk of all-cause mortality among mirtazapine users, compared to sertraline (aHR 1.16, 95% CI 1.05–1.29, *P* =0.004; Figure 3). Primary causes for death were similar between groups and comprised neoplasms, mental and behavioural disorders, and nervous, circulatory and respiratory system disorders among 80.3% (*n* = 1143) of deaths for mirtazapine and 80.1% (*n* = 427) for sertraline users (Supplementary Tables 2 and 3).

### Sensitivity analyses

The intention-to-treat estimates were of the same direction, strength and statistical significance (Supplementary Table 4). Most risk estimates were comparable at 90, 365 and 730 days, except for falls and fractures that were not statistically significant within 90 days (Supplementary Table 5). The addition of deaths records to ascertain cardiovascular events showed negligible differences.

## Discussion

This study among residents of LTCFs found mirtazapine use following LTCF entry was associated with a 16% higher risk of all-cause mortality, compared to sertraline, and a lower risk of falls and fractures after 90 days. There was no difference in risk of falls and fractures in the short-term (≤90 days), or cardiovascular-, dementia- or delirium-related hospitalisations during any period. These findings provide clinicians with new evidence about adverse outcomes that are important to older people living in LTCFs [36], to inform shared decisions with residents and their families when considering treatment options for depression.

There are no prior LTCF-specific studies examining the association between mirtazapine use and mortality. In the general population, a higher risk of mortality has been observed with mirtazapine use compared to no antidepressant use [19] or other antidepressant types or classes [19, 21, 37, 38]. However, individuals without dementia or aged ≥80 years had a lower risk of mortality compared to citalopram users [21]. Another study found adults (*n* = 25 598) who augmented their treatment for depression or switched from an SSRI to mirtazapine had a higher risk of all-cause mortality within 2 years compared to initiating another SSRI and a higher risk ≥2 years when compared to another SSRI, amitriptyline or venlafaxine [39]. There was also a higher risk of respiratory- and neoplasm-related mortality associated with mirtazapine compared to another SSRI within 2 years [39]. These were common reasons for death in our analysis. However, causes of death were similar between mirtazapine and sertraline users and there was no difference in risk of cardiovascular events. It is possible that unmeasured confounding or prescribing and deprescribing cascades post-LTCF entry influenced the higher risk of mortality associated with mirtazapine compared to sertraline. Further investigation is needed to understand cause-specific mortality risks. The 16% higher risk of all-cause mortality associated with mirtazapine is concerning given it is the fifth most common medicine supplied in Australian LTCFs [40] and received by one in five residents [1]. The potential population-level risk of harm associated with mirtazapine use in LTCFs is considerable and clinicians should consider this in the context of the limited evidence of its effectiveness [8, 9].

There was no statistically significant difference in risk of falls or fractures (short-term), cardiovascular-, dementia- or delirium-related hospitalisations between mirtazapine and sertraline use in LTCFs. This is in accordance with a study among 60 746 older people with depression, which reported a higher risk of stroke/transient ischemic attack, falls and fractures with mirtazapine and sertraline, versus no antidepressant use [19]. No prior studies have investigated the comparative risk of dementia-, delirium- or cardiovascular-related hospitalisations among mirtazapine or sertraline users in LTCFs. A higher risk of falls associated with SSRIs, but not with mirtazapine or other antidepressants, has been reported [41–43], with risk higher at treatment initiation or dose changes [43, 44]. A target trial emulation study following 101 953 older adults found mirtazapine and sertraline were associated with a lower risk of falls and related injuries in 1 year compared to no treatment (antidepressant or psychological therapy), and there were no differences in falls between these antidepressants [45]. Our study similarly found no short-term differences in falls or fractures between mirtazapine and sertraline. However, the observed lower risk of falls and fractures associated with mirtazapine compared to sertraline after 90 days, together with findings from LTCF studies [20, 41, 42, 44], suggests the underlying mechanism for falls and fractures in the longer-term may differ between these agents. This warrants further investigation, alongside the potential contribution of prescribing cascades, such as changes (e.g. co-prescribing or deprescribing) in use of other psychotropic or sedating medicines [43], and administration timing (e.g. mirtazapine taken at bedtime) [20] to the risk of antidepressant-related adverse outcomes in LTCFs.

There are important clinical factors not measured in this study that may influence antidepressant choice and the risk of adverse outcomes. These include untreated depression, changes in mood, behaviour, sleep or weight, sarcopenia, hyponatremia, orthostatic hypotension and sedation [20, 44, 46]. In LTCFs, mirtazapine is commonly prescribed at subtherapeutic doses for depression [5, 47] (i.e. where it is most sedating [8]), thus may be used for sedation rather than treatment of moderate–severe major depression. Minimal, mild and moderate depressive symptoms are experienced by 97% of new residents with depression [6]. Prescribers’ decisions to select one antidepressant over another are often guided by safety profiles and existing prescribing experience [48]. Residents’ low weight often drives preference for mirtazapine [7], as weight gain is a common side effect. Our study included residents initiating an antidepressant within 2 months of LTCF entry, which may not be sufficient time for residents’ new healthcare practitioners to screen, identify and diagnose moderate–severe major depression. It is likely that mirtazapine may be used for other indications (e.g. sleep, weight, low mood, symptoms of dementia), which are not supported by evidence for effectiveness [7–9]. The findings of this study highlight the risk of harm associated with mirtazapine use in LTCFs, providing new evidence to optimise person-centred, evidence-based therapeutic approaches to support resident mental health and wellbeing.

### Strengths and limitations

This is the first population-level study to examine risk of adverse outcomes associated with mirtazapine compared to sertraline in LTCFs. An active new user study design with propensity score adjustments was employed to minimise biases. Our linked data source facilitated comprehensive capture of potential confounders to be included in propensity score calculations and consideration for the competing risk of death, which is methodologically important in studies among residents of LTCFs, who are often approaching end-of-life [49]. Additionally, both as-treated and intention-to-treat approaches were conducted to rule out selection bias or exposure misclassification [25]. We included individuals entering LTCFs in three of Australia’s largest states (78% of Australia’s LTCF population [1]) and thus findings are likely nationally representative and internationally generalisable to countries with similar aged care systems.

Limitations include potential for unmeasured confounding, which could be related to clinical information around antidepressant administration, indication, doses, depression severity and sedation, which are not available in the datasets used. Under-ascertainment of health conditions is also possible when claims data are used. The time-varying nature of covariates could not be examined as many were obtained from care assessments conducted at care entry and not routinely repeated afterwards. Outcomes were measured from hospital, emergency and death data collections and represent more critical incidents. This study compared risk of adverse outcomes between two antidepressants and did not look at no antidepressant or nonpharmacological use due to data availability and low uptake of these services (<3% of residents receive government-subsidised mental health services [50]).

## Conclusions

This real-world study raises concern about the potential increased risk of harm associated with mirtazapine use in LTCFs, which is used by one in five residents. It supports the need for clinicians to consider safer alternatives in LTCFs and the place of antidepressants in treatment pathways, given 60% of residents use an antidepressant. This study highlights the need to regularly review and monitor antidepressant safety and effectiveness among residents using these medicines and to consider discontinuation and/or nonpharmacological alternatives where appropriate.

## Supplementary Material

aa-24-2498-File002_afaf074
