# Correction to: Risk of adverse outcomes associated with mirtazapine compared to sertraline use among older people living in long-term care facilities

**DOI:** 10.1093/ageing/afaf178

**Published:** 2025-06-13

This is a correction to: Georgina A Hughes, Maria C Inacio, Debra Rowett, Gillian E Caughey, Tracy Air, Catherine E Lang, Megan Corlis, Janet K Sluggett, Risk of adverse outcomes associated with mirtazapine compared to sertraline use among older people living in long-term care facilities, *Age and Ageing*, Volume 54, Issue 4, April 2025, afaf074, https://doi.org/10.1093/ageing/afaf074

The following changes have been made to the originally published manuscript. The affiliations have been emended for the second and third co-authors to read: ``[...] Maria C. Inacio^2,3,4^, Debra Rowett^1,5^[...]'' instead of: ``[...] Maria C. Inacio^3,4^, Debra Rowett^5^[...]''.

