# Supplementary material for: Risk of adverse outcomes associated with mirtazapine compared to sertraline use among older people living in long-term care facilities
Source: Age Ageing. 2025 Apr 6;54(4):afaf074. doi: 10.1093/ageing/afaf074 (PMC11972633; doi:10.1093/ageing/afaf074)
Supplement: aa-24-2498-File002_afaf074 [file aa-24-2498-file002_afaf074.docx]

**SUPPLEMENTARY INFORMATION**

**Supplementary Table 1.** Ascertainment of outcomes and health conditions in datasets.

**Supplementary Table 2.** Top ten main causes of death among individuals who died during follow-up, by antidepressant type.

**Supplementary Table 3.** Causes of death

**Supplementary Table 4.** Number of individuals experiencing adverse outcomes and associated risk among residents initiating mirtazapine versus sertraline, using the intention-to-treat approach (sensitivity analysis).

**Supplementary Table 5.** Risk of adverse outcomes overall and within 90 days, one and two years, among residents initiating mirtazapine versus sertraline.

**Supplementary Figure 1.** Diagram of study entry and follow-up.

**Supplementary Figure 2.** Cumulative incidence of fractures and the competing risk of death among the weighted cohorts, by antidepressant type.

**Supplementary Figure 3.** Cumulative incidence of cardiovascular events and the competing risk of death among the weighted cohorts, by antidepressant type.

**Supplementary Figure 4.** Cumulative incidence of dementia-related hospitalisations and the competing risk of death among the weighted cohorts, by antidepressant type.

**Supplementary Figure 5.** Cumulative incidence of delirium-related hospitalisations and the competing risk of death among the weighted cohorts, by antidepressant type.

**Supplementary Table 1.** Ascertainment of outcomes and health conditions in datasets.

| **Outcome** | **Description** | |
| --- | --- | --- |
| All-cause mortality  Definition  Dataset | Date of death  National Death Index | |
| Fall  Definition  Datasets  Defined by | Fall-related hospitalization, where the onset was not during the hospitalisation, or death from fall.  Hospital and ED claims, National Death Index  ROSA Outcome Monitoring System | |
| Fracture  Definition  Datasets  Defined by | Fracture-related hospitalisation caused by a fall, or secondary diagnosis for hospital/ED presentation for fracture, where the onset was not during the hospitalisation, claim for treatment of a fracture, or death from fracture.  Hospital and ED claims, MBS, National Death Index  ROSA Outcome Monitoring System | |
| Cardiovascular event  Definition  Datasets  Defined by  Transient Ischaemic Attack  Stroke  Myocardial infarction | Hospitalisation or presentation to the ED, where principal diagnosis (determined by ICD-10-AM code) was for transient ischaemic attack, stroke or myocardial infarction.  Ascertained from hospital and ED claims, and a sensitivity analysis also included if recorded as reason for death (by ICD-10-AM codes below).  ICD-10-AM codes  G45.0–G45.2, G45.8, G45.9  I60.0-160.9. I61.0-I61.9 (exc I61.7), I62.0, I62.1, I62.9, I63.0-I63.9 (exc I63.7), I64  I21.0-I21.4, I21.9, I22.0, I22.1, I22.8, I22.9, I23.0-I23.6, I23.8 | |
| Dementia-related hospitalization  Definition  Datasets  Defined by | Primary reason for hospitalisation or ED presentation was dementia  Hospital and ED claims  ROSA Outcome Monitoring System | |
| Delirium-related hospitalization  Definition  Datasets  Defined by | Primary reason for hospitalisation or ED presentation was delirium  Hospital and ED claims  ROSA Outcome Monitoring System | |
| **Health condition** | **Aged care assessments and health condition codes** | |
| Dementia | Aged care eligibility assessment  Entry to care assessment | HC_0500-0504, HC_0510-0516, HC_0520-0526, HC_0530-0532, HC_0584  HC_0500 HC_0510 HC_0520 HC_0530 |
| Depression | Aged care eligibility assessment  Entry to care assessment | HC_0550, HC_0552  HC_0550A |
| Osteoporosis | Aged care assessment data | HC_1306 |
| Diabetes | Aged care assessment data | HC_0402, HC_0403, HC_0404 |
| Malnutrition | Aged care assessment data | HC_0405 |
| History of falls | Aged care assessment data | HC_1715 |
| History of fractures | Aged care assessment data | HC_1606-1612 |
| History of delirium | Aged care eligibility assessment  Entry to care assessment | HC_0540-0544  HC_0540 |
| Transient Ischemic Attack | Aged care assessment data | HC_0605 |
| Stroke | Aged care assessment data | HC_0911, HC_0912, HC_0913, HC_0914, HC_0915 |
| Myocardial infarction | Aged care assessment data | HC_0904 |

MBS, Medicare Benefits Schedule, ROSA, Registry of Senior Australians, ED, emergency department, ICD-10-AM, International Classification of Diseases Tenth Revision Australian Modification, exc, excluding. Hospital and ED claims in New South Wales, Victoria and Queensland were analysed.

**Supplementary Table 2.** Causes of death among individuals during follow-up (n=1,957) by antidepressant type.

| **ICD-10-AM chapter group**^†^ | **Mirtazapine users (n=1,424)*** | **Sertraline users (n=533)*** |
| --- | --- | --- |
| Certain infectious and parasitic diseases e.g., sepsis, gastroenteritis and colitis [A00-B99] | 30 (2.1) | 17 (3.2) |
| Neoplasms e.g., malignant neoplasms of lung, prostate [C00-D49] | 238 (16.7) | 86 (16.1) |
| Endocrine, nutritional and metabolic disorders e.g., fluid, electrolyte and acid-base balance disorders, type 2 diabetes mellitus and complications, volume depletion, malnutrition [E00-E90] | 66 (4.6) | 18 (3.4) |
| Mental and behavioural disorders e.g., delirium and/or dementia, depressive episode, anxiety disorder [F00-F99] | 166 (11.7) | 65 (12.2) |
| Diseases of the nervous system e.g., Alzheimer’s disease, Parkinson’s disease, transient cerebral ischaemic attack, Lewy body disease [G00-G99] | 140 (9.8) | 51 (9.6) |
| Diseases of the blood and blood-forming organs and certain disorders involving the immune mechanism; or congenital malformations, deformations and chromosomal abnormalities; Symptoms, signs and abnormal clinical and laboratory findings not elsewhere classified; or diseases of the skin; or diseases of the musculoskeletal system e.g., anaemia, tendency to fall, malaise and fatigue, cognitive function/awareness, cellulitis, ulcer, back pain, osteoporosis with fracture [D50-D89; Q00-Q99; R00-R99; L00-L99; M00-M99] | 35 (2.5) | 10 (1.9) |
| Diseases of the circulatory system e.g., heart failure, ischaemic stroke, myocardial infarction, arrhythmias, orthostatic hypotension [I00-I99] | 436 (30.6) | 177 (33.2) |
| Diseases of the respiratory system e.g., pneumonia, respiratory infection, chronic obstructive pulmonary disease, influenza [J00-J99] | 163 (11.4) | 48 (9.0) |
| Diseases of the digestive system e.g., intestinal obstructions, diverticulitis [K00-K95] | 49 (3.4) | 23 (4.3) |
| Diseases of the genitourinary system e.g., urinary tract infection, acute kidney failure, chronic kidney disease [N00-N99] | 46 (3.2) | 11 (2.1) |
| Injury, poisoning and certain other consequences of external causes; or external causes of morbidity and mortality; or special purposes  e.g., fractures, traumatic subdural haemorrhage, traumatic ischemia of muscle [S00-T88; V01-Y99; W01, W06, W19, W80; X590, X599, X61] | 43 (3.0) | 22 (4.1) |

ICD-10-AM, International Classification of Diseases Tenth Revision Australian Modification.

*Does not include n=70 individuals with missing data for determining propensity score (complete case analysis).

†There were 17 (0.9%) individuals who died and were missing cause of death.

**Supplementary Table 3.** Top ten main causes of death during follow-up (n=1,957), by antidepressant type.

|  | **Mirtazapine users (n=1,424)*** | | **Sertraline users (n=533)*** | |
| --- | --- | --- | --- | --- |
|  | **ICD-10-AM code^†^** | **n (%)** | **ICD-10-AM code^†^** | **n (%)** |
| **1** | F03 (unspecified dementia) | 141 (9.9) | F03 (unspecified dementia) | 48 (9.0) |
| **2** | G309 (Alzheimer’s disease, unspecified) | 70 (4.9) | I259 (chronic ischemic heart disease, unspecified) | 31 (5.8) |
| **3** | I259 (chronic ischemic heart disease, unspecified) | 70 (4.9) | I219 (acute myocardial infarction, unspecified) | 27 (5.1) |
| **4** | I219 (acute myocardial infarction, unspecified) | 68 (4.8) | I64 (stroke) | 25 (4.7) |
| **5** | I64 (stroke) | 64 (4.5) | G309 (Alzheimer’s disease, unspecified) | 19 (3.6) |
| **6** | C349 (malignant neoplasm of bronchus or lung, unspecified) | 40 (2.8) | C349 (malignant neoplasm of bronchus or lung, unspecified) | 14 (2.6) |
| **7** | J449 (chronic obstructive pulmonary disease, unspecified) | 39 (2.7) | G20 (Parkinson’s disease) | 14 (2.6) |
| **8** | I489 (atrial fibrillation and atrial flutter, unspecified) | 33 (2.3) | F019 (vascular dementia, unspecified) | 13 (2.4) |
| **9** | C61 (malignant neoplasm of prostate) | 31 (2.2) | I509 (heart failure, unspecified) | 13 (2.4) |
| **10** | J440 (chronic obstructive pulmonary disease with acute lower respiratory tract infection) and G20 (Parkinson’s disease)^‡^ | 28 (2.0) | J440 (chronic obstructive pulmonary disease with acute lower respiratory tract infection) | 12 (2.3) |

ICD-10-AM, International Classification of Diseases Tenth Revision Australian Modification.

*Does not include n=70 individuals with missing data for determining propensity scores (complete case analysis).

†There were 17 (0.9%) individuals who died and were missing cause of death.

‡There was the same number of mirtazapine users with J440 and G20 recorded as their main cause of death (n=28).

**Supplementary Table 4.** Number of individuals experiencing adverse outcomes and associated risk among residents initiating mirtazapine versus sertraline, using the intention-to-treat approach (sensitivity analysis).

| **Outcome** | **Crude (n,%)** | | **Weighted* (n,%)** | | **Weighted hazard ratio (aHR) (95% CI, p)*** | **Subdistribution hazard ratio (aSHR)**  **(95% CI, p)*** |
| --- | --- | --- | --- | --- | --- | --- |
|  | *Mirtazapine (n=3,837)* | *Sertraline (n=1,572)* | *Mirtazapine (n=3,711)* | *Sertraline (n=1,517)* |  |  |
| Fall  Overall  ≤90 days  >90 days | 1 032 (26.9)  323 (8.4)  709 (18.5) | 483 (30.7)  135 (8.6)  348 (22.1) | 994 (26.8)  310 (8.4)  684 (18.4) | 474 (31.2)  127 (8.4)  347 (22.9) | **0.88 (0.78-0.98), 0.020**  1.02 (0.83-1.26), 0.832  **0.82 (0.72-0.94), 0.004** | **0.85 (0.76-0.95), 0.004**  1.01 (0.82-1.24), 0.939  **0.79 (0.69-0.90), <0.001** |
| Fracture  Overall  ≤90 days  >90 days | 500 (13.0)  115 (3.0)  385 (10.0) | 251 (16.0)  52 (3.3)  199 (12.7) | 481 (13.0)  109 (2.9)  372 (10.0) | 250 (16.5)  49 (3.2)  200 (13.2) | **0.81 (0.69-0.94), 0.007**  0.92 (0.66-1.30), 0.648  **0.78 (0.65-0.94), 0.007** | **0.78 (0.67-0.91), 0.002**  0.91 (0.65-1.28), 0.581  **0.75 (0.62-0.90), 0.002** |
| Cardiovascular event | 244 (6.4) | 103 (6.6) | 242 (6.5) | 95 (6.3) | 1.10 (0.86-1.40), 0.443 | 1.07 (0.81-1.40), 0.643 |
| Dementia | 155 (4.0) | 69 (4.4) | 153 (4.1) | 70 (4.6) | 0.93 (0.70-1.25), 0.636 | 0.90 (0.67-1.20), 0.474 |
| Delirium | 270 (7.0) | 101 (6.4) | 251 (6.8) | 100 (6.6) | 1.08 (0.85-1.37), 0.536 | 1.04 (0.82-1.33), 0.738 |
| All-cause mortality | 2 266 (59.1) | 911 (58.0) | 2 214 (59.7) | 876 (57.7) | **1.09 (1.01-1.18), 0.037** | n/a |

CI, confidence interval, n/a not applicable. Statistically significant results are shown in bold.

*Inverse probability treatment weighted (IPTW) cohort does not include n=182 individuals with missing data for determining propensity scores (complete case analysis).

**Supplementary Table 5.** Risk of adverse outcomes overall and within 90 days, one and two years, among residents initiating mirtazapine versus sertraline.

| **Outcome** | **Weighted hazard ratio (aHR) (95% CI, p)*** | | | | **Subdistribution hazard ratio (aSHR)**  **(95% CI, p)*** | | | |
| --- | --- | --- | --- | --- | --- | --- | --- | --- |
|  |  |  |  |  |  |  |  |  |
| **Risk window** | **Overall** | **90 days** | **365 days** | **730 days** | **Overall** | **90 days** | **365 days** | **730 days** |
| Fall | **0.86 (0.75-0.97), 0.018** | 1.01 (0.81-1.25), 0.961 | 0.87 (0.75-1.01), 0.070 | **0.85 (0.74-0.98), 0.020** | **0.82 (0.72-0.94), 0.003** | 0.99 (0.80-1.23), 0.939 | **0.85 (0.73-0.99). 0.034** | **0.82 (0.72-0.94), 0.005** |
| Fracture | **0.74 (0.62-0.89), 0.001** | 0.94 (0.67-1.34), 0.739 | **0.76 (0.60-0.95), 0.015** | **0.74 (0.61-0.89), 0.002** | **0.71 (0.59-0.85), <0.001** | 0.93 (0.66-1.31), 0.674 | **0.74 (0.59-0.92), 0.008** | **0.71 (0.59-0.85), <0.001** |
| Cardiovascular event | 1.11 (0.84-1.45), 0.459 | 1.05 (0.65-1.70), 0.837 | 1.01 (0.73-1.39), 0.977 | 1.08 (0.81-1.42), 0.614 | 1.06 (0.81-1.39), 0.684 | 1.04 (0.64-1.67), 0.885 | 0.98 (0.71-1.35), 0.887 | 1.03 (0.78-1.37), 0.833 |
| Dementia | 1.03 (0.74-1.44), 0.862 | 1.41 (0.84-2.37), 0.193 | 1.09 (0.74-1.60), 0.663 | 1.10 (0.77-1.56), 0.613 | 1.00 (0.71-1.39), 0.975 | 1.39 (0.83-2.34), 0.210 | 1.07 (0.73-1.56), 0.742 | 1.06 (0.75-1.51), 0.739 |
| Delirium | 1.22 (0.92-1.62), 0.172 | 1.53 (0.98-2.40), 0.063 | 1.32 (0.94-1.84), 0.110 | 1.21 (0.90-1.63), 0.200 | 1.17 (0.88-1.56), 0.267 | 1.51 (0.96-2.37), 0.072 | 1.29 (0.92-1.80), 0.142 | 1.17 (0.87-1.58), 0.288 |
| All-cause mortality | **1.16 (1.05-1.29), 0.004** | **1.24 (1.02-1.49), 0.029** | **1.18 (1.04-1.34), 0.010** | **1.19 (1.07-1.33), 0.002** | n/a | n/a | n/a | n/a |

CI, confidence interval, n/a, not applicable. Statistically significant results are shown in bold.

*Inverse probability treatment weighted (IPTW) cohort does not include n=182 individuals with missing data for determining propensity scores (complete case analysis).

**Supplementary Figure 1.** Diagram of study entry and follow-up.


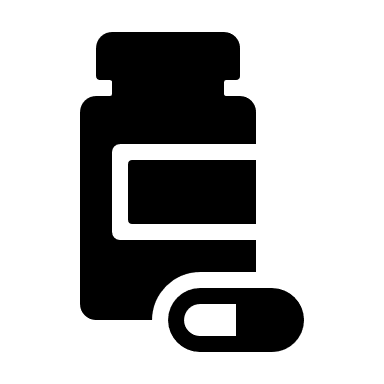

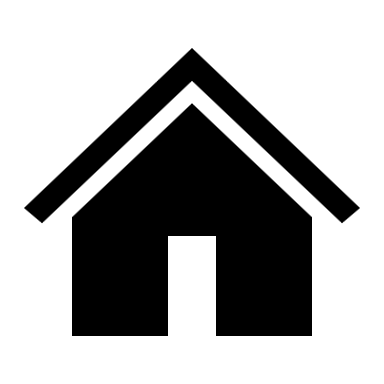


LTCF entry

01/01/2015-31/10/2018

First dispensing of mirtazapine or sertraline while accessing care, between LTCF entry and ≤60 days afterwards (index date)

*No dispensing of any antidepressant 120 days prior to LTCF entry*

*Individuals were followed from index date until the outcome of interest, antidepressant discontinuation, dispensing of another antidepressant, exit from LTCF, death or 31 December 2019 (whichever occurred first).*

**60 days**

**120 days**

**Up to 1 year before LTCF entry**

*Information for covariates*

31/12/2019

LTCF, long-term care facility.

**Supplementary Figure 2.** Cumulative incidence of fractures and the competing risk of death among the weighted cohorts, by antidepressant type.

Number

at risk:

Overall 5228 2041 917 368 113

Sertraline 1517 602 278 110 32

Mirtazapine 3711 1440 640 258 81

Cumulative incidence of fractures, and death as the competing event, is plotted. Blue line at 90 days for time-varying effects. Plot truncated to 4 years due to low numbers at risk thereafter. Numbers at risk of the inverse probability of treatment weighting (IPTW) cohort are listed by exposure group at bottom of plot.

**Supplementary Figure 3.** Cumulative incidence of cardiovascular events and the competing risk of death among the weighted cohorts, by antidepressant type.

Number

at risk:

Overall 5228 2154 1007 419 131

Sertraline 1517 651 315 126 39

Mirtazapine 3711 1503 692 293 92

Cumulative incidence of cardiovascular events, and death as the competing event, is plotted. Plot truncated to 4 years due to low numbers at risk thereafter. Numbers at risk of the inverse probability of treatment weighting (IPTW) cohort are listed by exposure group at bottom of plot.

**Supplementary Figure 4.** Cumulative incidence of dementia-related hospitalisations and the competing risk of death among the weighted cohorts, by antidepressant type.

Number

at risk:

Overall 5228 2183 1044 431 140

Sertraline 1517 663 334 134 45

Mirtazapine 3711 1519 710 297 94

Cumulative incidence of dementia-related hospitalisations, and death as the competing event, is plotted. Plot truncated to 4 years due to low numbers at risk thereafter. Numbers at risk of the inverse probability of treatment weighting (IPTW) cohort are listed by exposure group at bottom of plot.

**Supplementary Figure 5.** Cumulative incidence of delirium-related hospitalisations and the competing risk of death among the weighted cohorts, by antidepressant type.

Number

at risk:

Overall 5228 2138 1020 415 136

Sertraline 1517 654 325 126 40

Mirtazapine 3711 1485 694 289 95

Cumulative incidence of delirium-related hospitalisations, and death as the competing event, is plotted. Plot truncated to 4 years due to low numbers at risk thereafter. Numbers at risk of the inverse probability of treatment weighting (IPTW) cohort are listed by exposure group at bottom of plot.
